# Shared Decision Making Does Not Influence Physicians against Clinical Practice Guidelines

**DOI:** 10.1371/journal.pone.0062537

**Published:** 2013-04-24

**Authors:** Mireille Guerrier, France Légaré, Stéphane Turcotte, Michel Labrecque, Louis-Paul Rivest

**Affiliations:** 1 Research Center of the Centre Hospitalier Universitaire de Québec, Québec, Québec, Canada; 2 Faculty of Medicine, Department of Family Medicine and Emergency Medicine, Université Laval, Québec, Québec, Canada; 3 Faculty of Sciences and Engineering, Department of Mathematics and Statistics, Université Laval, Québec, Québec, Canada; University of Maryland, United States of America

## Abstract

**Background:**

While shared decision making (SDM) and adherence to clinical practice guidelines (CPGs) are important, some believe they are incompatible. This study explored the mutual influence between physicians’ intention to engage in SDM and their intention to follow CPGs.

**Methods:**

Embedded within a clustered randomized trial to assess the impact of training physicians in SDM about using antibiotics to treat acute respiratory tract infections, this study evaluated physicians’ intentions to both engage in SDM and follow CPGs. A self-administered questionnaire based on the theory of planned behavior evaluated both behavioral intentions and their respective determinants (attitude, subjective norm and perceived behavioral control) at study entry and exit. We used path analysis to explore the relationships between the intentions. We conducted statistical analyses using the maximum likelihood method and the variance-covariance matrix. Goodness of fit indices encompassed the chi-square statistic, the comparative fit index and the root mean square error of approximation.

**Results:**

We analyzed 244 responses at entry and 236 at exit. In the control group, at entry we observed that physicians’ intention to engage in SDM (r = 0, t = 0.03) did not affect their intention to follow CPGs; however, their intention to follow CPGs (r = −0.31 t = −2.82) did negatively influence their intention to engage in SDM. At exit, neither behavioral intention influenced the other. In the experimental group, at entry neither behavioral intention influenced the other; at exit, the intention to engage in SDM still did not influence the intention to use CPGs, although the intention to follow CPGs (r = −0.15 t = −2.02) slightly negatively influenced the intention to engage in SDM, but this was not clinically significant.

**Conclusion:**

Physicians’ intention to engage in SDM does not affect their intention to adopt CPGs even after SDM training. Physicians’ intention to adopt CPGs had no clinically significant influence on intention to engage in SDM.

**Trial Registration:**

ClinicalTrials.gov NCT01116076

## Introduction

Implementing shared decision making (SDM) and the recommendations of clinical practice guidelines (CPG) in clinical practice are both high-priority items on the healthcare policy-making agendas of industrialized countries [1], [2]. Application of SDM and CPGs in practice should both improve patient outcomes and promote effective, equitable and rational utilization of resources [3], [4]. These two approaches are however quite different.

SDM is an approach in which health-related decision making process is made jointly by the patient and his/her healthcare provider(s) [5], [6]. SDM incorporates the principles of both patient-centered care and evidence-based medicine: by sharing the information on the benefits and risks of all available options with the patient, communicating probabilistic nature of evidence and eliciting and discussing the patient’s values and preferences, the health professional helps the patient making an informed decision, the goal of SDM [4], [7], [8].

CPGs are “systematically developed statements to assist practitioner and patient decisions about appropriate health care for specific clinical circumstances” [9]. Guidelines developed using the GRADE system [10], which has the backing of numerous respected organizations such as the World Health Organization [11], distinguish between treatments for which benefits clearly outweigh risks and those for which they are more closely balanced for a population. A CPG that makes this distinction can indicate to physicians when they need to help patients weigh up the desirable and undesirable effects carefully according to their values and preferences. Other organizations (e.g. the American Thoracic Society) recommend integrating patient preferences into the process itself of CPG development [12]. However, there is evidence that this is not occurring [13], [14].

In spite of these initiatives, most CPGs do not discuss the importance of patient values and preferences in therapeutic decision making and few provide information about benefits and risks as well as the probabilistic nature of evidence in a way that fosters the process of SDM [14], [15]. The “do or don’t” style of recommendation is still preponderant even when the strength of the recommendations is graded.

Little is known about translating SDM and CPGs together into clinical practice and it is unclear whether physicians are likely to adopt both at once [16], [17]. In fact stakeholders have expressed concerns that the implementation of SDM and CPGs in clinical practice at the same time will complicate both. The development of, access to, and training for implementing both CPG and SDM and related tools, such as patient decision aids, are very uneven. Contrasting theoretical stances about treatment decisions may also make SDM and CPGs difficult to reconcile. Integrating the evidence with patient preferences requires individualizing the benefit/harm trade-off. A problem arises when doctors are told to implement evidence-based guidelines without individualizing that evidence and without incorporating patient preferences. Furthermore, government-imposed practice incentives based on CPGs may ride roughshod over patient choice [18]. From a behavioral perspective, physicians who adopt one clinical behavior (e.g. adhering to CPGs) could be motivated by practice incentives that impede the adoption of the other behavior (e.g. engaging in SDM).

Our conceptual underpinning for assessing the relationship between the two behaviors (adopting CPGs and engaging in SDM) was the theory of planned behavior (TPB), a theory that identifies intention as the principal predictor of the realization of behavior [19]. Intention can be predicted with high accuracy by measuring its three main determinants: attitude, subjective norm and perceived behavioral control. *Attitude* refers to being favorably or unfavorably disposed towards the behavior based on its potential outcomes. *Subjective norm* refers to perceived social pressure to perform the behavior or not. *Perceived behavioral control* refers to the perception of hindering or facilitating factors, and reflects personal resources. Evidence from meta-analyses and systematic reviews suggests that the TPB has greater predictive performance of behavioral intention and performance than other socio-cognitive theories [20].

In an earlier pilot study assessing the feasibility of a larger trial, we explored the relationship between physicians’ intention to follow CPGs and their intention to engage in SDM [21], [22]. Results from this exploratory work suggested that adherence to CPGs and engagement in SDM are independent intentions that have no effect on each other (unpublished data; data available by contacting authors). However, this exploratory work was limited by its small sample size (only 30 physicians). Hence we used data from the larger clustered randomized trial in which this study is embedded to confirm or contest our earlier findings that there was no mutual influence between the intentions to engage in SDM and to follow CPGs.

### Hypothesis

Based on an exploratory study and theoretical considerations [19], [21], we had identified the link between each intention (SDM and CPG) and its three predictive determinants. We hypothesized the existence of an association between the two intentions in the context of a clinical encounter where both are measured at the same time. We expected to observe how determinants of intention to engage in SDM affect intention to follow CPG and how determinants of intention to follow CPG affect intention to participate in SDM.

## Materials and Methods

### Ethics Statement

The review boards of the two institutions involved, the Centre de Santé et de Services Sociaux de la Vieille-Capitale and the Centre de Santé et de Services Sociaux du Nord de Lanaudière, approved the study. We obtained written informed consent from all participants.

### Study Design

This study consisted of secondary analyses of data obtained from a multi-center, two-arm, parallel cluster randomized trial conducted in a network of the 12 family practice teaching units (units of randomization) affiliated with the Department of Family Medicine and Emergency Medicine at Université Laval in six different regions of the Province of Quebec, Canada. The design, methods, and findings of this trial have been described elsewhere [21]–[23]. The trial was conducted in three phases: a) baseline data collection (physician and patient recruitment) from July through October 2010; b) intervention (training of physicians in the experimental group in DECISION+2, a training program in SDM) in November 2010, and c) post-intervention data collection (patient recruitment) from November 2010 through April 2011.

The experimental group received a two-hour online tutorial followed by a two-hour on-site workshop. They also received a decision support tool. The training program addressed key elements of the clinical decision-making process concerning antibiotic treatment for acute respiratory tract infections (ARTIs) in primary care and how physicians could share the information and the decision with their patients. A detailed description of the DECISION+2 training has been published elsewhere [23]. The program also informed them of CPGs produced by the Institut National d’Excellence en Santé et Services Sociaux (INESSS) of the Province of Quebec about first and second choices for antibiotics to treat ARTIs [24]. These CPGs are well-known among Quebec family physicians [25]. Physicians in the control group provided routine care during the trial period and had no access to the online tutorial, the on-site interactive workshop or the decision support tool.

### Participants

We recruited all physicians–teachers and residents–who provided care in one of the nine family practice teaching units (out of 12) that enrolled in the study. We excluded physicians who participated in the pilot trial [21], [22] and those who would not be practicing at the family practice teaching unit during the trial (e.g. residents ending their residency program, physicians doing rotations outside the family practice teaching unit, physicians who expected to be pregnant and physicians planning to retire).

### Data Collection Procedures

At entry to and exit from the study, we collected secondary outcomes in all participating physicians that included: a) their intention to engage in SDM in future consultations regarding the use of antibiotics for ARTIs and b) their intention to follow any CPGs regarding the use of antibiotics in the context of ARTI.

Based on the TPB model, our self-administered questionnaire assessed the two behavioral intentions with a 7-point Likert scale ranging from −3 (strongly disagree) to +3 (strongly agree). It also assessed the determinants of these intentions as defined by the TPB: we measured attitude using six questions and we measured subjective norm and perceived behavioral control using three questions each [26], [27]. When items for these constructs were subjected to factor analysis, a model with three factors was found to consistently represent both intentions. Two items loaded on perceived behavioral control (alpha Cronbach ≥0.5), three on subjective norm (alpha Cronbach ≥0.9), and four on attitude (alpha Cronbach ≥0.8). We removed other items from the analysis. All models at study entry and exit presented good fit of indices [28] (Appendix S1, Appendix S2 and Appendix S3). Sociodemographic information was recorded at trial entry for physicians.

### Statistical Analyses

**Figure 1:**
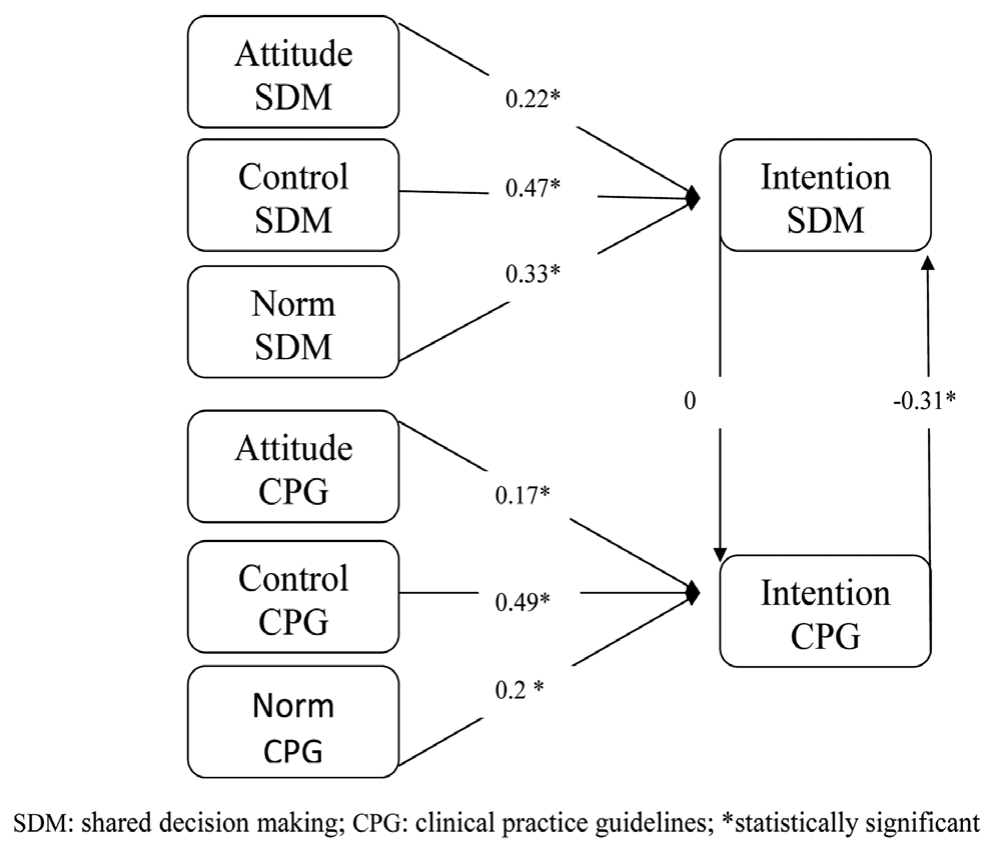
Standardized path analysis coefficients for control group at study entry, showing attitude, perceived behavioral control and subjective norm as predictors of physicians’ behavioral intentions to engage in SDM and to follow CPGs; and showing the influence of the intention to engage in SDM on the intention to follow CPGs and vice versa (vertical arrows).

**Figure 2:**
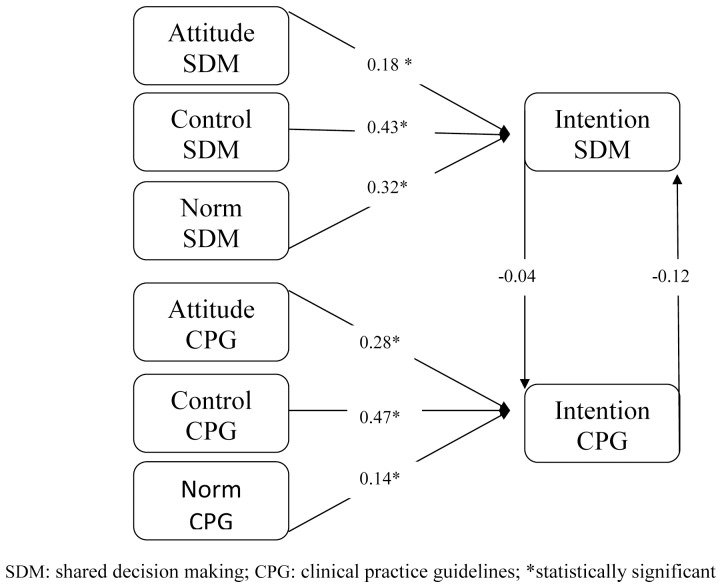
Standardized path analysis coefficients for experimental group at study entry, showing attitude, perceived behavioral control and subjective norm as predictors of physicians’ behavioral intentions to engage in SDM and to follow CPGs; and showing the influence of the intention to engage in SDM on the intention to follow CPGs and vice versa (vertical arrows).

We performed simple descriptive statistics including means and standard deviation for the study variables to summarize physicians’ clinical characteristics. Incomplete data were removed from the data set. We implemented structural equation models with path analysis that allowed us to test hypothetical associations in sample data [29]. We used path analysis to assess the association between the two behavioral intentions. In path analysis, a standardized path coefficient can be interpreted as a correlation measure that shows the effect of an independent variable on a dependent variable. A direct effect is illustrated by an arrow pointing directly at the dependent variable, while we defined indirect effects as effects that are transmitted via intervening variables. Thus, the intention itself to engage in SDM has a *direct effect* on the intention to follow CPGs, in addition to predicting its behavioral determinants, while the three predicted determinants of the intention to engage in SDM have *indirect effects* on physicians’ intention to follow CPGs. This same pattern of direct and indirect effects applies to the intention to engage in SDM (Figures 1 and 2). We used means of the intentions and their three respective determinants (attitude, subjective norm and perceived behavioral control) to adjust a non-recursive model. All analyses were conducted using maximum likelihood method and the variance-covariance matrix. Goodness of fit indices obtaining for different models encompassed the chi-square statistic with degrees of freedom, the comparative fit index or CFI [30] and the root mean square error of approximation, or RMSEA. Where chi-square divided by the degrees of freedom was less than 2, CFI values were over 0.9, and RMSEA was less than 0.60, the fits were considered acceptable [28], [31]–[33]. Next, we used the path coefficient to test the significance and measure the direction (positive or negative) and extent of the influence between the two intentions. The statistical results of the path coefficients are presented as pairs (r, t) where r, a standardized coefficient, can be interpreted as a correlation measure, and t is a normal statistic distribution that tests whether a path coefficient is null. Values of t that are larger than 2 in absolute value correspond to path coefficients that are significantly non-null at p equal to 0.05 level. We used AMOS Software and the SAS statistical package for performing the analyses.

## Results

### Participants

We collected questionnaires from 250 participating physicians at study entry and 269 at exit. After removing incomplete data, we analyzed 244 responses (out of 250) at entry and 236 (out of 242) at exit. Because missing data was minimal (less than 5%) and results with imputed data led to very similar results, we decided not to present results with imputed data [34]. Physician characteristics are presented in Table 1.

**Table 1:** Characteristics of participants.

	Control	Experimental
PHCPs, n	108	161
*Participating teachers,* n/N (%)	53/108 (49)	78/161 (48)
Women, n/N (%)	36/53 (68)	49/78 (63)
Mean±SD years of age	44±10	42±9
Mean±SD years of professional experience	15±11	14±10
*Residents*, n/N (%)	55/108 (51)	83/161 (52)
Women, n/N (%)	34/55 (68)	60/83 (72)
Mean±SD years of age	27±4	28±5

SD: standard deviation; PCHPs: primary care health providers.

### Means and Standard Deviations of the Measures Assessed

Descriptive statistics of mean scores of physician intentions and the determinants of those intentions are presented in Table 2. At study entry, physician scores for intention to follow CPGs were higher than their scores for intention to engage in SDM, a pattern similar in both study groups and matched by the scores for the determinants of each intention. At study exit, we observed a similar relationship between the intentions and their respective determinants.

**Table 2:** Mean scores for constructs.

Constructs	Modalityof Scale		Mean±SD						
			Entry					Exit	
			Control			Experimental		Control	Experimental
	**SDM**								
Intention	(−3, 3)		1.7±0.9	1.7±0.8				1.8±0.7	1.7±0.9
Attitude			1.2±1.1	1.4±0.9				1.5±1.1	1.7±0.8
Norm			1.4±1	1.5±0.9				1.7±0.8	1.6±0.9
Control			1.6±1	1.4±1				1.5±1	1.5±1
	**CPG**								
Intention	(−3, 3)	2.3±0.7			2.2±0.6		2.3±0.6		2.1±0.7
Attitude		1.8±1.1			1.8±0.9		1.8±1		1.8±0.9
Norm		2±0.9			2±0.8		2±0.8		2±0.8
Control		2.2±0.9			2±0.9		2±0.8		1.8±0.9

SD: standard deviation; SDM: shared decision making; CPG: clinical practice guidelines.

### Relationship between Intention to follow CPGs and Intention to Engage in SDM

**Figure 3:**
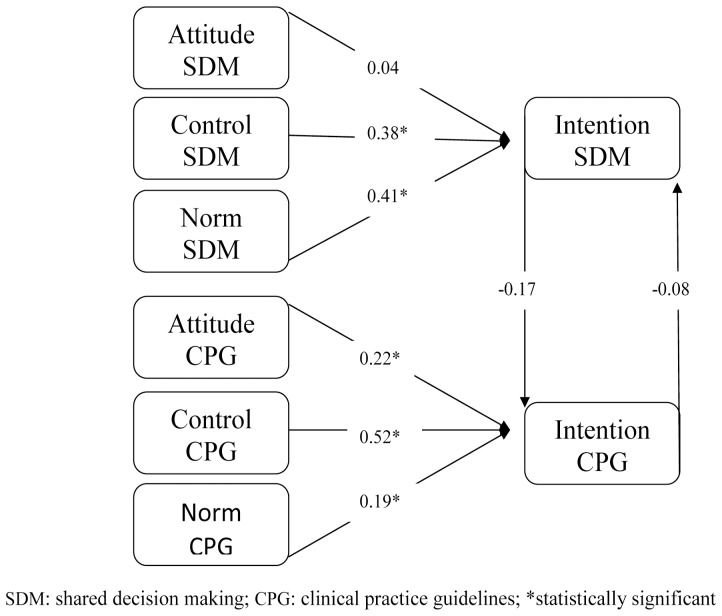
Standardized path analysis coefficients for control group at study exit, showing attitude, perceived behavioral control and subjective norm as predictors of physicians’ behavioral intentions to engage in SDM and to follow CPGs; and showing the influence of the intention to engage in SDM on the intention to follow CPGs and vice versa (vertical arrows).

**Figure 4:**
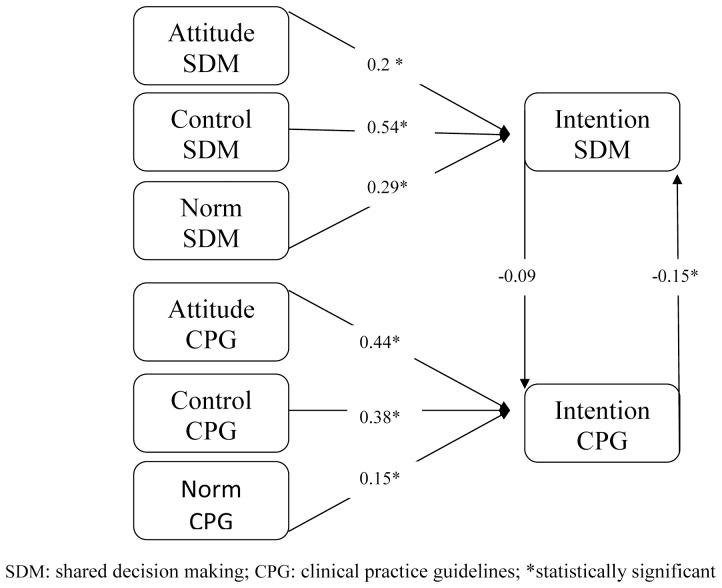
Standardized path analysis coefficients for experimental group at study exit, showing attitude, perceived behavioral control and subjective norm as predictors of physicians’ behavioral intentions to engage in SDM and to follow CPGs; and showing the influence of the intention to engage in SDM on the intention to follow CPGs and vice versa (vertical arrows).

Path analyses indicated a good fit for all models (see Table 3). All standardized path coefficients (see Figures 1, 2, 3 and 4) of intention to engage in SDM or intention to follow CPGs, including attitude, subjective norm and perceived behavioral control, were considered significant at p<0.05.

**Table 3:** Goodness of fit indices for the path analysis.

Model	Chi-square	DF	pvalue	Chi-square/df^a^	RMSEA^b^	CFI^c^
**Entry**						
Control	1.4	4	0.3	1.2	0	1
Experimental	4.3	4	0.4	1.1	0	1
**Exit**						
Control	1.3	4	0.9	0.3	0	1
Experimental	2.1	4	0.7	0.5	0	1

DF: degree of freedom; RMSEA: root mean square error of approximation; CFI: comparative fit index;

a chi-square/df <2 indicated a good fit of model;

b RMSEA <0.06 indicated a goof fit of model;

c CFI>0.9 indicated a good fit of model.

At entry into the study in the control group, physicians’ intention to engage in SDM (r = 0, t = 0. 03) did not affect their intention to follow CPGs; however, their intention to follow CPGs (r = −0.31 t = −2.82) did negatively influence their intention to engage in SDM. Once they had left the study, there was still no significant influence of the intention to engage in SDM (r = −0.17 t = −1.48) on the intention to follow CPGs, and moreover the intention to follow CPGs (r = −0.08 t = −1.71) no longer negatively affected the intention to engage in SDM (figures 1 and 3).

At entry into the study in the experimental group, physicians’ intention to engage in SDM (r = −0.04 t = −0.45) had no influence on their intention to follow CPGs, nor did their intention to follow CPGs (r = −0.12, t = −1.32) influence their intention to engage in SDM; at exit, their intention to engage in SDM still did not influence their intention to use CPGs, although their intention to follow CPGs (r = −0.15 t = −2.02) did negatively influence their intention to engage in SDM but this was not clinically significant (figures 2 and 4).

### Direct and Indirect Effects of the Determinants of Intention

Each of the determinants of intention defined as direct effects, i.e. attitude, subjective norm and perceived behavioral control, significantly affected both intentions (follow CPGs and engage in SDM). At study entry, perceived behavioral control was the primary factor determining intentions in both groups (r = 0.49 and 0.47 for CPGs, r = 0.47 and 0.43 for SDM, respectively in control and experimental groups). Nevertheless, at exit, we observed that in the control group the most important determining factor of the intention to engage in SDM shifted to subjective norm. Also, in the experimental group, attitude was the most important factor determining the intention to use CPGs.

At entry into the control group, the intention to engage in SDM was affected indirectly by two behavioral factors of the intention to follow CPGs. We observed small negative indirect effects of perceived control (r = −0.15 t = −2.9) and norm (r = −0.06 t = −2.1) on the intention to engage in SDM but this was not clinically significant. These two correlations are statistically different from zero. None of the other indirect effects was statistically significant (Table 4). At study exit, there was no indirect effect of any predictive determinant through the intervening intention.

**Table 4:** Indirect effects on each intention.

	Entry		Exit	
	Control	Experimental	Control	Experimental
**Indirect effects on SDM intention**				
Attitude CPG	−0.05	−0.03	−0.02	−0.06
Control CPG	−0.15*	−0.06	−0.04	−0.06
Norm CPG	−0.06*	−0.02	−0.02	−0.02
**Indirect effects on CPG intention**				
Attitude SDM	0	−0.01	0	−0.02
Control SDM	0	−0.02	−0.07	−0.05
Norm SDM	0	−0.01	−0.07	−0.03

SDM: shared decision making; CPG: clinical practice guidelines;

* statistically significant at 5%.

## Discussion

To the best of our knowledge, few studies have used social cognitive theories to understand physicians’ simultaneous adoption of any two clinical behaviors [35], [36], and none have studied specifically the combined adoption of engaging in SDM and following CPGs. Our study addresses this gap. In addition, our study measured how the relationship between the two behaviors changed over a period of time. In the control group, at study entry we found that physicians’ intention to follow CPGs negatively affected their intention to engage in SDM, while the reverse was not true. By the time they left the study, neither intention had any significant influence on the other. In the experimental group, at entry into the study physicians’ intention to follow CPGs had no influence on their intention to engage in SDM, and vice versa. At study exit, their intention to engage in SDM still had no influence on their intention to follow CPGs, although their intention to follow CPGs had a slight negative effect on their intention to engage in SDM but this was not clinically significant. Our results lead us to make three main observations.

First, some descriptive studies report physicians’ concerns that SDM may not be compatible with adhering to CPGs. One of these studies reports that physician feel compelled to choose one over the other [37], [38]. Another suggests that rigidly applying guidelines can limit patient choice and may damage the doctor-patient relationship by preventing physicians from responding to patients’ needs and preferences [38]–[40]. We observed a slight influence of the intention to use CPGs on the intention to practice SDM, but this influence was not clinically significant. Our results show we should not be concerned that physicians who integrate one into their clinical practice will not integrate the other. Physicians showed high scores for intention to integrate both CPGs and SDM, a pattern that did not change over time in both study groups. This is valuable evidence for partisans of both SDM and CPGs. Our findings lend evidence-based support to a current of thinking that promotes the accommodation of patient preferences in CPGs to improve the quality of decision making [41]. They also support standards for trustworthy guidelines that promote the consideration of patient preferences as appropriate [42], [43]. CPGs should not only identify decisions for which patient preferences are important (as facilitated by the GRADE system), but could also provide recommendations about how to communicate benefits/harms of options, assess values and preferences, and include tools such as decision aids to facilitate the SDM process [44].

Second, our study provides parties interested in implementing SDM and adhering to CPGs at the same time with theory-based information regarding what strategies may be effective for both. The results of a systematic review of the use of socio-cognitive models to explain behavior change among healthcare professionals concluded that the TPB is an appropriate model for predicting behavior [45]. Applying this theory to behavioral intentions we consistently found that at study entry, among both study groups, perceived behavioral control (perception of barriers and facilitators to changing their behavior) and subjective norm (perceived social pressure to perform the behavior or not) were the most important factors determining the intention to engage in SDM, and perceived behavioral control was the most important factor determining the intention to follow CPGs. For implementation purposes, this suggests that addressing the barriers physicians perceive to adopting these behaviors is more relevant than, say, addressing the disposition of the physician (attitude) towards adopting them. Furthermore, the barriers themselves must be addressed individually, as they differ for each intention. Earlier studies assessing perceived barriers to SDM found those most often mentioned were time constraints, lack of agreement and perceived lack of applicability due to patient characteristics or clinical situations [46], while studies assessing a variety of barriers to using CPGs generally specify lack of awareness of CPGs, lack of familiarity and lack of agreement [47]. While healthcare managers and educators need to address these barriers individually for both clinical behaviors, generic and less costly approaches could be designed to address the physicians’ perceived behavioral control regarding each behavior, the meaningful determining factor in both cases.

Third, the before-after measures in our study suggest that the relationship between the theory-based variables proposed by the TPB and the behavioral intention may change over time. After SDM training, we observed that perceived behavioral control and subjective norm were still the most important variables predicting physicians’ intention to engage in SDM, but their intention to follow CPGs, in the experimental group, was mostly predicted by perceived behavioral control and attitude (being favorably or unfavorably disposed towards the behavior based on its potential outcomes). This finding regarding determinants of intention is congruent with one study showing that increased physician intention (based on the theoretical model) to adopt the Medline System for practicing evidence-based medicine was particularly determined by their attitude toward that system [48]. This seems to suggest that exposing physicians to an SDM training program might improve their adherence to CPGs. Further studies would be needed to confirm our findings regarding this changing relationship.

Our study has a few limitations. First, we acknowledge that it was embedded in a larger study and not designed specifically to assess the mutual influence of physician intentions to engage in SDM and to follow CPGs. Second, we cannot infer that our results represent the behavioral intentions of physicians in other clinical context and settings. Our study was conducted in academically-oriented clinical settings, and physicians practicing in FPTUs are not representative of the whole population of health professionals [49], [50]. In terms of secondary analyses, our study was limited by the sample size in each study group and we were unable to guarantee enough power for our statistical analyses. Lastly, we did not assess the performance of the behaviors of interest (subsequent to expressions of intention) using objective measures such as third-observer assessment. A systematic review of the application of socio-cognitive theories to explain clinical behaviors among health professional has reported an intention-behavior gap [45]. This study was not designed to address this gap and future studies should consider including objective measures of both behaviors subsequent to intention.

## Conclusion

Intention to adopt SDM regarding the use of antibiotics to treat ARTIs does not negatively influence physicians’ intention to use CPG recommendations in the same clinical context, including after exposure to a training program in SDM. There is no clinically significant influence of the intention to use CPGs on the intention to practice SDM. In recent years, concerns have been raised that healthcare practitioners’ engagement in SDM may interfere with their following CPGs, and vice versa [18]. This study shows that at this point in time, there is no evidence to justify these concerns.

## Supporting Information

Appendix S1 **Theory of planned behavior model.**
(TIF)

Appendix S2 **Confirmatory analyses.**
(DOCX)

Appendix S3 **List of items.**
(DOCX)
