# Supplementary figures and images for: Shared Decision Making Does Not Influence Physicians against Clinical Practice Guidelines
Source: PLoS One. 2013 Apr 24;8(4):e62537. doi: 10.1371/journal.pone.0062537 (PMC3634782; doi:10.1371/journal.pone.0062537)

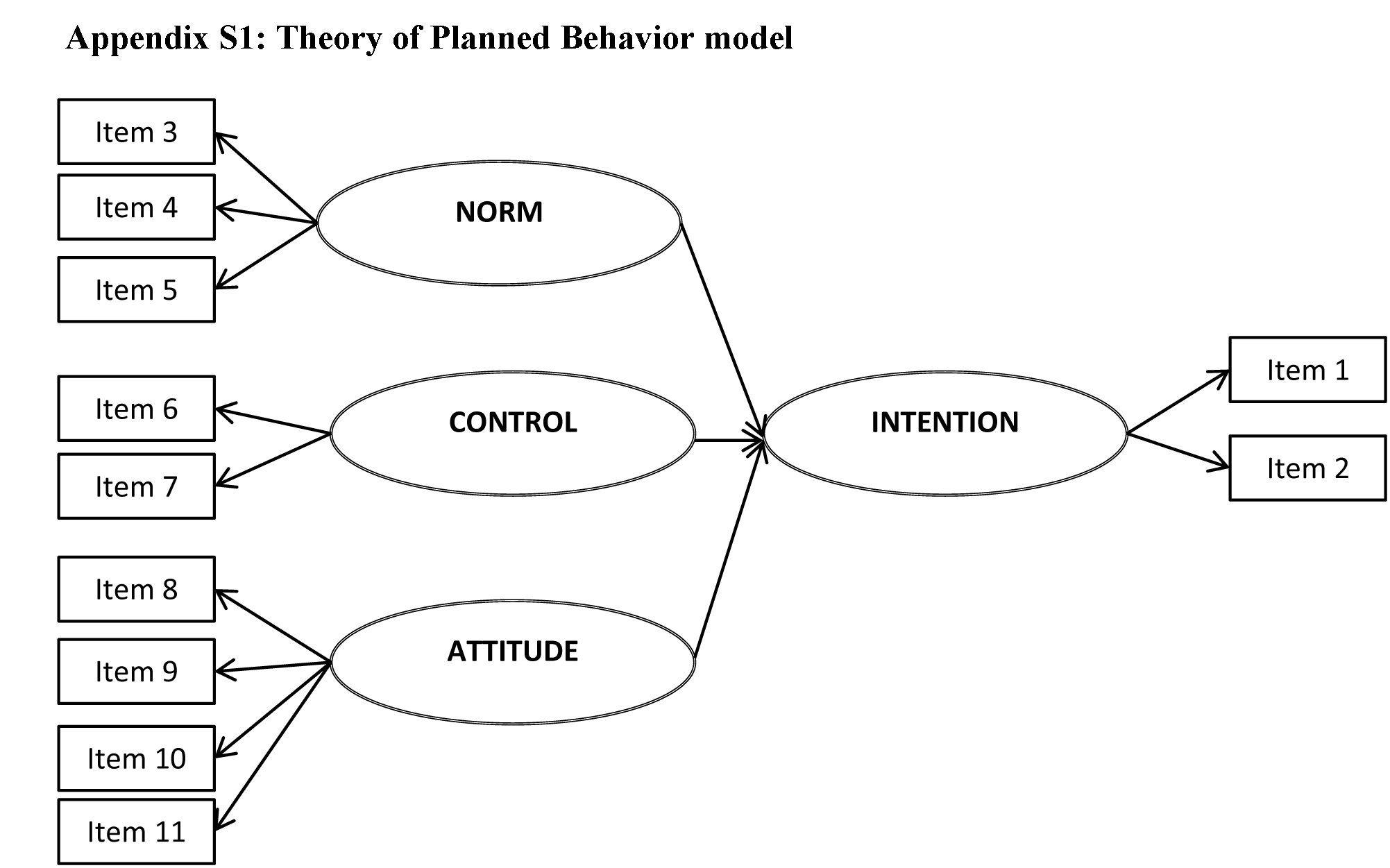

Supplement: Appendix S1 — Theory of planned behavior model. (TIF) [file pone.0062537.s001.tif]
