# Supplementary material for: Shared Decision Making Does Not Influence Physicians against Clinical Practice Guidelines
Source: PLoS One. 2013 Apr 24;8(4):e62537. doi: 10.1371/journal.pone.0062537 (PMC3634782; doi:10.1371/journal.pone.0062537)
Supplement: Appendix S2 — Confirmatory analyses. (DOCX) [file pone.0062537.s002.docx]

**Appendix S2: Confirmatory analyses**

**Table: Goodness of fit indices for confirmatory factor analysis**

| **Model** | **Chi-square** | **DF** | **Chi-square/df**^a^ | **RMSEA**^b^ | **CFI**^c^ |
| --- | --- | --- | --- | --- | --- |
| Intention SDM at entry | 81.72 | 37 | 2.21 | 0.07 | 0.97 |
| Intention SDM at exit | 67.01 | 38 | 1.76 | 0.06 | 0.98 |
| Intention CPG at entry | 53.54 | 37 | 1.45 | 0.04 | 0.99 |
| Intention CPG at exit | 70.08 | 36 | 1.95 | 0.06 | 0.98 |

Notes: DF: degree of freedom; RMSEA: root mean square error of approximation; CFI: comparative fit index; ^a^ chi-square/df <2 indicated a good fit of model; ^b^ RMSEA <0.06 indicated a goof fit of model; ^c^ CFI>0.9 indicated a good fit of model
