# Supplementary material for: Shared Decision Making Does Not Influence Physicians against Clinical Practice Guidelines
Source: PLoS One. 2013 Apr 24;8(4):e62537. doi: 10.1371/journal.pone.0062537 (PMC3634782; doi:10.1371/journal.pone.0062537)
Supplement: Appendix S3 — List of items. (DOCX) [file pone.0062537.s003.docx]

**Appendix S3: List of items**

|  | **Intention SDM** |
| --- | --- |
| 1 | When a decision needs to be made about antibiotic prescription during a consultation with a patient presenting with an acute respiratory infection. what are the chances that you would engage in shared decision making? …. Very low–Very high |
| 2 | When a decision needs to be made about antibiotic prescription during a consultation with a patient presenting with an acute respiratory infection. I intend to engage in shared decision making … Strongly disagree–Strongly agree |
|  | **Norm SDM** |
| 3 | Most of the people who are important to me would recommend that I engage in shared decision making when a decision needs to be made about antibiotic prescription during a consultation with a patient presenting with an acute respiratory infection … Strongly disagree–Strongly agree |
| 4 | Most of the people who are important to me would think it preferable that I engage in shared decision making when a decision needs to be made about antibiotic prescription during a consultation with a patient presenting with an acute respiratory infection … Strongly disagree–Strongly agree |
| 5 | Most of the people who are important to me are favorable that I engage in shared decision making when a decision needs to be made about antibiotic prescription during a consultation with a patient presenting with an acute respiratory infection … Strongly disagree–Strongly agree |
|  | **Control SDM** |
| 6 | I don’t see any obstacles to engaging in shared decision making when a decision needs to be made about antibiotic prescription during a consultation with a patient presenting with an acute respiratory infection … Strongly disagree–Strongly agree |
| 7 | I feel I would be capable of engaging in shared decision making when a decision needs to be made about antibiotic prescription during a consultation with a patient presenting with an acute respiratory infection … Strongly disagree–Strongly agree |
|  | **Attitude SDM** |
| 8 | When a decision needs to be made about antibiotic prescription during a consultation with a patient presenting with an acute respiratory infection. I think engaging in shared decision making would be … Very irresponsible–Very responsible |
| 9 | When a decision needs to be made about antibiotic prescription during a consultation with a patient presenting with an acute respiratory infection. I think engaging in shared decision making would be … Very unpleasant–Very pleasant |
| 10 | When a decision needs to be made about antibiotic prescription during a consultation with a patient presenting with an acute respiratory infection. I think engaging in shared decision making would make me feel … Worthless–Very worthwhile |
| 11 | When a decision needs to be made about antibiotic prescription during a consultation with a patient presenting with an acute respiratory infection. I think engaging in shared decision making would be … Very annoying–Very gratifying |

|  | **Intention CPG** |
| --- | --- |
| 1 | When a decision needs to be made about antibiotic prescription during a consultation with a patient presenting with an acute respiratory infection. What are the chances that you would adopt clinical practice guidelines? …. Very low–Very high |
| 2 | When a decision needs to be made about antibiotic prescription during a consultation with a patient presenting with an acute respiratory infection. I intend to adopt the clinical practice guidelines … Strongly disagree–Strongly agree |
|  | **Norm CPG** |
| 3 | Most of the people who are important to me would recommend that I adopt the clinical practice guidelines when a decision needs to be made about antibiotic prescription during a consultation with a patient presenting with an acute respiratory infection … Strongly disagree–Strongly agree |
| 4 | Most of the people who are important to me would think it preferable that I adopt the clinical practice guidelines when a decision needs to be made about antibiotic prescription during a consultation with a patient presenting with an acute respiratory infection … Strongly disagree–Strongly agree |
| 5 | Most of the people who are important to me are favorable that I adopt the clinical practice guidelines when a decision needs to be made about antibiotic prescription during a consultation with a patient presenting with an acute respiratory infection … Strongly disagree–Strongly agree |
|  | **Control CPG** |
| 6 | I don’t see any obstacles to adopting the clinical practice guidelines when a decision needs to be made about antibiotic prescription during a consultation with a patient presenting with an acute respiratory infection … Strongly disagree–Strongly agree |
| 7 | I feel I would be capable of adopting the clinical practice guidelines when a decision needs to be made about antibiotic prescription during a consultation with a patient presenting with an acute respiratory infection … Strongly disagree–Strongly agree |
|  | **Attitude CPG** |
| 8 | When a decision needs to be made about antibiotic prescription during a consultation with a patient presenting with an acute respiratory infection. I think adopting the clinical practice guidelines would be … Very irresponsible–Very responsible |
| 9 | When a decision needs to be made about antibiotic prescription during a consultation with a patient presenting with an acute respiratory infection. I think adopting the clinical practice guidelines would be … Very unpleasant–Very pleasant |
| 10 | When a decision needs to be made about antibiotic prescription during a consultation with a patient presenting with an acute respiratory infection. I think adopting the clinical practice guidelines would make me feel … Worthless–Very worthwhile |
| 11 | When a decision needs to be made about antibiotic prescription during a consultation with a patient presenting with an acute respiratory infection. I think adopting the clinical practice guidelines would be … Very annoying–Very gratifying |
